# The oral–gut–liver axis: linking periodontal microbiota to the pathogenesis of liver diseases

**DOI:** 10.3389/fmed.2026.1810114

**Published:** 2026-04-23

**Authors:** Fagui Wu, Yuxin Chen, Xiuqin Ni, Tingzhuang Yi, Shengkui Tan

**Affiliations:** 1 Guangxi Clinical Medical Research Center for Hepatobiliary Diseases, The Affiliated Hospital of Youjiang Medical University for Nationalities, Baise, Guangxi, China; 2 Guangxi Higher Education Research Center for Gastrointestinal Microecology and Health Engineering, The Affiliated Hospital of Youjiang Medical University for Nationalities, Baise, Guangxi, China; 3 Baise City Key Laboratory of Gastrointestinal Microecology and Health, The Affiliated Hospital of Youjiang Medical University for Nationalities, Baise, Guangxi, China

**Keywords:** hepatocellular carcinoma, microbiome, non-alcoholic fatty liver disease, oral–gut–liver axis, periodontitis

## Abstract

Oral microbiota plays a critical role in linking oral and systemic health, with dysbiosis closely associated with the onset and progression of chronic liver diseases. This review systematically examines the central role of the “oral–gut–liver axis” in hepatic pathophysiology. Epidemiological evidence has identified periodontitis and specific oral pathogens, such as *Fusobacterium nucleatum* (*F. nucleatum*), as independent risk factors for the progression of non-alcoholic fatty liver disease (NAFLD), development of cirrhosis, and incidence of hepatocellular carcinoma (HCC). The underlying mechanisms primarily involve four interrelated pathways: (1) direct bacterial translocation, where pathogens such as *F. nucleatum* colonize the liver via bacteremia and activate oncogenic pathways; (2) systemic dissemination of bacterial metabolites, such as lipopolysaccharides (LPS), driving hepatic inflammation, oxidative stress, and fibrosis via Toll-like receptor 4 (TLR4) signaling and reactive oxygen species (ROS)-mediated pathways; (3) systemic immune inflammation, wherein periodontitis acts as a chronic inflammatory focus that continuously releases pro-inflammatory mediators into the circulation; and (4) indirect effects mediated by gut microbiota dysbiosis, whereby oral bacteria compromise the intestinal barrier, facilitating the influx of gut-derived toxins into the liver. These findings underscore the significant impact of oral health on hepatic status. In the short term, oral microbial profiles represent promising noninvasive diagnostic and prognostic biomarkers. Preliminary clinical trials indicate that periodontal therapy can improve metabolic parameters in patients with NAFLD. In the long term, promoting interdisciplinary collaboration between hepatology and oral medicine and strategically integrating oral health interventions into the comprehensive management framework for liver diseases hold significant public health potential for mitigating the global burden of hepatic disorders.

## 1 Introduction

### 1.1 Chronic liver diseases

Chronic liver diseases, including non-alcoholic fatty liver disease (NAFLD) and hepatocellular carcinoma (HCC), pose significant global public health challenges. NAFLD is the most prevalent chronic liver disease worldwide, affecting approximately 25% of the global population. Its progressive form, non-alcoholic steatohepatitis (NASH), can progress to liver fibrosis, cirrhosis, and ultimately HCC (1). In China, it is estimated that over one-fifth of the population is affected by various chronic liver diseases (CLDs), notably cirrhosis, HCC, NAFLD, and alcoholic liver disease (ALD), which are induced by environmental and biological factors (2). CLDs and their complications are leading causes of substantial global morbidity and mortality. Despite the increasing disease burden, current therapeutic strategies remain limited. No universally effective pharmacotherapy is available for NASH, and treatments for advanced HCC often demonstrate restricted efficacy and considerable adverse effects (3). This highlights the urgent need to investigate novel pathogenic mechanisms, identify modifiable risk factors, and discover early diagnostic biomarkers for liver diseases. Central to the pathogenic effects of periodontal bacteria on the liver is the interplay between inflammation and oxidative stress. Reactive oxygen species play a critical role in activating inflammatory signaling cascades—particularly the NF-κB and NLRP3 inflammasome pathways—while simultaneously inflicting direct damage on hepatocyte DNA, proteins, and lipids. Nearly all chronic inflammatory conditions, including periodontitis and chronic liver diseases, share oxidative damage as a core pathological component. Periodontal pathogens such as *P. gingivalis* and *F. nucleatum* can induce ROS production both locally within the periodontium and systemically following translocation to the liver, thereby establishing a vicious cycle in which inflammation promotes oxidative stress and oxidative stress, in turn, amplifies inflammation (4).

In this review, we synthesize current evidence to propose that oral dysbiosis influences hepatic pathophysiology through four principal mechanisms that often intersecting with direct bacterial translocation, metabolite-mediated toxicity, systemic immune-inflammatory activation, and gut microbiota-mediated indirect effects.

### 1.2 Human microbiome and systemic diseases

Over the past decade, breakthroughs in human microbiome research have unveiled its central role in maintaining host homeostasis and driving the pathogenesis of diseases. Initial investigations predominantly focused on the gut microbiome, elucidating its key functions in nutrient metabolism, immune regulation, and barrier integrity, and establishing a strong association with various systemic conditions, including obesity, diabetes, and autoimmune disorders (5). Subsequent research has recognized the oral microbiota as one of the most diverse and abundant microbial communities in the human body, forming a highly complex microecosystem. This system engages in crosstalk with distant organs through mechanisms such as bacteremia, immunomodulation, and metabolite signaling. Substantial evidence has linked oral dysbiosis to systemic diseases, including cardiovascular disease, diabetes, adverse pregnancy outcomes, and neurodegenerative disorders, indicating that the ecological equilibrium of the oral microbiota plays a critical role in sustaining systemic health (6). The pathogenesis of periodontitis involves complex interactions between bacterial infection and host immune responses (7). Pathogenic shifts in the oral microbial community can induce not only local inflammation but also a state of systemic inflammation. Disruption of oral microbial homeostasis precipitates excessive immune activation, facilitating the development of oral diseases. More significantly, this dysbiosis can affect systemic health through multiple pathways, contributing to the pathogenesis of conditions such as diabetes, cardiovascular disease, and NAFLD. For instance, studies have demonstrated that *F. nucleatum* originating from the oral cavity of patients with periodontitis can translocate to the liver via the bloodstream, where it delivers virulence factors such as lipopolysaccharide (LPS) to activate Toll-like receptor 4 (TLR4) on Kupffer cells, subsequently triggering the downstream nuclear factor kappa B (NF-κB) -mediated inflammatory cascade (8, 9). This activation triggers the myeloid differentiation primary response 88 (MyD88)-dependent signaling pathway, leading to NF-κB transcription and the subsequent release of pro-inflammatory cytokines, such as interleukin-6 (IL-6) and tumor necrosis factor-alpha (TNF-*α*), thereby accelerating NASH progression. In addition to *F. nucleatum*, several other periodontal pathogens have been implicated in the pathogenesis of liver disease. The “red complex” pathogens—*Porphyromonas gingivalis*, *Tannerella forsythia*, and *Treponema denticola*—are strongly associated with chronic periodontitis and have been identified as potential risk factors for liver cirrhosis (10). *Aggregatibacter actinomycetemcomitans*, another key periodontal bacterium, has been shown to affect NAFLD by altering gut microbiota composition and glucose metabolism. This is supported by clinical studies in NAFLD patients and by animal experiments demonstrating that administration of *A. actinomycetemcomitans* impairs glucose tolerance and exacerbates hepatic steatosis (11). *Prevotella intermedia* is also among the periodontal pathogens capable of translocating from the oral cavity to the intestine, potentially contributing to liver pathology. Importantly, the oral-liver axis is not limited to a single pathogenic species; rather, it involves complex, community-level microbial changes in which multiple periodontal bacteria synergistically contribute to hepatic inflammation and fibrosis (12). A cohort study of 10,245 patients further corroborated this link, demonstrating that individuals with chronic periodontitis had a 1.7-fold higher risk of developing cirrhosis than healthy controls (HR = 1.7, 95% CI 1.2–2.4) (13). The precise threshold for oral pathogen translocation to distant organs has not yet been defined, and targeted interventions leveraging the “oral-liver axis” require validation through large-scale clinical trials. Future studies should employ multi-omics technologies to decipher the spatiotemporal dynamics linking oral microbial metabolite and hepatic disease progression.

### 1.3 Proposing the “oral–gut–liver axis” hypothesis

Given this context, the liver, an organ that receives portal venous blood and is continuously exposed to gut-derived microbes and their products, is a key target for investigating the distal effects of the oral microbiota. In recent years, the concept of the “oral–gut–liver axis” has emerged and gained broad acceptance. This hypothesis posits that oral pathogens or their metabolites can enter the systemic circulation or gastrointestinal tract via mechanisms such as bacteremia and saliva ingestion, thereby systemically modulating the liver’s immune and metabolic milieu and playing a significant role in the pathogenesis of liver diseases (14, 15). Oral-derived pathogens, such as *Porphyromonas gingivalis* (*P. gingivalis*) and *F. nucleatum*, have been identified in both experimental models and clinical samples and are correlated with NAFLD severity and HCC development (16). Oral dysbiosis contributes to NAFLD progression, primarily by disrupting hepatic metabolism through interconnected pathways. The close anatomical and functional connection between the liver and gut, known as the gut-liver axis, is established via the portal vein, bile duct, and systemic circulation (17). Saliva ingestion introduces oral bacteria into the gastrointestinal tract. Among these, *F. nucleatum* not only colonizes the gut but also facilitates the colonization of other pathogenic species in the oral niche, thereby compromising intestinal barrier integrity (18). This dysbiosis facilitates the translocation of endotoxins, such as LPS, into the liver, where they activate the TLR4/NF-κB pathway in Kupffer cells and upregulate pro-inflammatory factors such as TNF-*α*. Furthermore, the liver’s unique anatomical position, with its dual blood supply from the systemic and portal circulations, allows periodontium-derived toxic factors to disseminate hematogenously (14). In alcohol-induced liver disease mouse models, colonization or infection with *P. gingivalis* aggravates liver injury. This effect is characterized by more pronounced hepatic inflammation, steatosis, and fibrosis, suggesting a novel extra-intestinal mechanism by which this oral pathogen exacerbates liver disease via the remote modulation of gut microbiota and host immunity (19). Additionally, LPS from periodontal pathogens (e.g., *Actinomyces* and *P. gingivalis*) can bind to TLR-4 and TLR-2 on immune cells. Concurrently, other bacterial components engage Toll-like receptors, activating intracellular signaling pathways that promote the expression of co-stimulatory molecules, such as CD86, on antigen-presenting cells. The subsequent CD86-CD28 interaction is instrumental in CD4 + T cell activation (20).

Consequently, establishing the oral microbiota as an overlooked but critical distal regulator of hepatic pathophysiology introduces a novel research framework in this field.

This review aims to systematically and critically evaluate the current research progress on the role of oral microbiota in liver diseases. We first summarize the epidemiological evidence supporting its association with NAFLD, cirrhosis, and HCC. Subsequently, we delve into the underlying multi-layered biological mechanisms, including direct bacterial translocation, metabolite effects, systemic inflammation, and indirect effects mediated by alterations in the gut microbiota. Finally, this review focuses on the translational implications of this field, including the potential of oral microbes as diagnostic biomarkers and the clinical prospects of intervening in liver disease by improving oral health. It also highlights current research challenges and future directions, aiming to provide a theoretical basis and novel perspectives for advancing this interdisciplinary field.

## 2 Epidemiological associations: from correlation to causal inference

### 2.1 Non-alcoholic fatty liver disease (NAFLD)

NAFLD is a highly prevalent chronic liver condition worldwide. Clinically, NAFLD is stratified into two principal phenotypes: non-alcoholic fatty liver (NAFL), characterized by simple steatosis, and non-alcoholic steatohepatitis (NASH), in which steatosis coexists with lobular inflammation and hepatocyte ballooning. NASH is a progressive form that frequently advances to liver fibrosis, which is a major determinant of liver-related mortality (21). The disease spectrum encompasses a continuum from simple steatosis and NASH to severe complications, including cirrhosis, end-stage liver disease, and HCC (22). The pathogenesis of NAFLD/NASH is multifactorial, involving complex interactions among altered energy metabolism, host immune responses, intestinal microbiota, and genetic susceptibility (13). Accumulating evidence underscores a significant association between NAFLD and dysbiosis of the oral and gut microbiota. Consequently, NAFLD is increasingly recognized not only as a hepatic disorder but also as a systemic metabolic condition characterized by an immune-metabolic network imbalance, with its pathogenesis intimately linked to disruptions along the oral–gut–liver axis (19). Established risk factors include obesity, insulin resistance, type 2 diabetes mellitus, dyslipidemia, and metabolic syndrome (23). Epidemiological studies have further identified a correlation between oral health and NAFLD; for instance, multiple cross-sectional analyses have reported a significant positive association between tooth loss and elevated NAFLD risk, indicating a dose–response relationship (24). Recent methodological advances, particularly Mendelian randomization, have helped to transcend the limitations of correlational studies by providing evidence for causal inference. One such study leveraging large-scale genomic data identified a positive causal relationship between genetic predisposition to periodontitis and increased NAFLD risk, suggesting that genetically influenced periodontal disease may contribute to NAFLD etiology, albeit potentially modulated by pleiotropic confounding factors (25).

Recent studies have demonstrated that periodontitis and chronic liver diseases share a common pathological basis, characterized by chronic inflammation and dysbiosis. The two conditions interact through mechanisms including microbial translocation, systemic immune activation, and gut microbiota dysregulation, forming a bidirectional causal relationship (26). On one hand, oral dysbiosis may promote liver injury through systemic inflammation, oral–gut microbial translocation, and endotoxemia. Virulence factors, such as lipopolysaccharide (LPS) produced by periodontal pathogens (particularly *Porphyromonas gingivalis*), can activate Toll-like receptor signaling pathways, trigger the release of NF-κB-mediated inflammatory cytokines, and induce oxidative stress and activation of hepatic Kupffer cells, thereby accelerating the progression of fatty liver disease, liver fibrosis, and hepatocellular carcinoma (12). The link between periodontitis and NAFLD is further supported by shared mechanisms of oxidative stress. Renu et al. systematically reviewed the evidence demonstrating that periodontopathic microbes exacerbate liver pathology through increased oxidative stress. ROS generated in response to periodontal infection activate the NF-κB and NLRP3 signaling pathways, leading to the release of pro-inflammatory cytokines such as IL-1β, IL-6, and TNF-*α*, which in turn promote hepatocyte injury, hepatic stellate cell activation, and fibrogenesis. Moreover, oxidative stress compromises intestinal barrier integrity, thereby facilitating the translocation of gut-derived endotoxins and further amplifying hepatic inflammation (4). On the other hand, advanced liver disease—particularly cirrhosis—increases the risk of severe periodontitis, primarily due to hepatic dysfunction-induced impairment of immune surveillance, reduced salivary antimicrobial factors, and alterations in the systemic metabolic and inflammatory milieu. These changes, in turn, promote oral dysbiosis and exacerbate periodontal inflammation. Furthermore, in patients with viral hepatitis, hepatitis B virus (HBV) and hepatitis C virus (HCV) markers have been detected in gingival crevicular fluid, suggesting that the oral cavity may serve as a viral reservoir—further corroborating the bidirectional relationship between these two conditions (27). Therefore, understanding this bidirectional relationship carries important clinical implications for establishing interdisciplinary collaboration between hepatology and oral medicine and for integrating oral health interventions into comprehensive management strategies for liver diseases.

These findings highlight the need for further investigation to delineate the specific pathogenic mechanisms, elucidate the interactions between oral bacteria and NAFLD, and ultimately inform the development of novel therapeutic strategies.

### 2.2 Liver fibrosis and cirrhosis

At the advanced end of the chronic liver disease spectrum, the association between oral health and the progression to liver fibrosis and decompensated cirrhosis is noteworthy. Research confirms that patients with cirrhosis commonly exhibit significant oral dysbiosis, characterized by a distinct salivary microbiota composition that differs from that of healthy individuals. The role of specific periodontal pathogens in cirrhosis has been further elucidated by clinical investigations. A study examining six periodontal bacterial species in patients with liver disease demonstrated that those with cirrhosis had a significantly increased risk of presenting with high counts of “red complex” bacteria (*P. gingivalis*, *T. forsythia*, and *T. denticola*). Multivariate analysis identified low albumin level (<3.7 g/dL), liver cirrhosis, and *P. gingivalis* fimA genotype II as independent risk factors associated with elevated red complex bacterial counts, with adjusted odds ratios of 6.93, 4.72, and 4.08, respectively (*p* < 0.05) (10). These findings suggest that patients with cirrhosis may require enhanced oral health care compared to those without cirrhosis to mitigate the risk of periodontal disease progression and its potential systemic consequences. These oral microbial alterations are synchronous with and correlated with shifts in the gut microbiota, suggesting that the oral cavity may serve as an indicator of intestinal microecological disturbances and reflects a close link between oral ecology and hepatic functional reserve (15). Observational studies have further underscored the clinical relevance of this association. A cross-sectional analysis of patients with cirrhosis identified a significant positive association between periodontal disease and cirrhosis severity. Periodontal disease is highly prevalent in this population, and its severity may correlate with more advanced liver disease and an elevated risk of complications, such as hepatic encephalopathy (28). Notably, mechanistic insights from rodent models have demonstrated that oral inoculation with *Porphyromonas gingivalis* can inhibit intestinal Farnesoid X receptor (FXR) signaling and, via activation of the TLR4/NF-κB pathway, increase hepatic triglyceride accumulation by 42%. These findings indicate that *P. gingivalis* induces concurrent disturbances in hepatic and intestinal ecology, which are pertinent to glucose/lipid metabolism and inflammation (29).

Collectively, the evidence indicates that periodontitis is significantly associated with an increased risk of cirrhosis. The underlying mechanism likely involves the continuous systemic dissemination of oral-derived microbes and inflammatory mediators, which exacerbates pre-existing systemic inflammation and immune dysregulation, thereby synergistically accelerating liver fibrosis progression and decompensation events (17).

### 2.3 Hepatocellular carcinoma

Recent research has established that specific oral pathogens can directly colonize HCC tissues and contribute to malignant progression. Multiple studies employing 16S rRNA gene sequencing, metagenomics, or qPCR have detected a significant enrichment of *F. nucleatum* within *HCC* tumor tissues, whereas its abundance was minimal in adjacent non-tumor liver tissue or healthy controls (30). This tumor-specific enrichment has important clinical implications. Prognostically, a high intratumoral burden of *F. nucleatum* serves as an independent risk factor for reduced overall and recurrence-free survival in patients, strongly supporting its potential utility as a prognostic biomarker (31). Mechanistically, *F. nucleatum* can bind to E-cadherin on hepatocytes via its surface adhesin FadA, thereby activating the *β*-catenin signaling pathway, which drives the transcription of proto-oncogenes and promotes aberrant hepatocyte proliferation (32). Furthermore, *F. nucleatum* remodels the tumor immune microenvironment through additional virulence factors; for instance, its Fap2 protein binds to the inhibitory receptor TIGIT (T cell immunoreceptor with Ig and ITIM domains) on natural killer (NK) cells and T cells, directly suppressing their antitumor activity and facilitating immune evasion (33).

Epidemiological evidence from large-scale cohort studies, including a decades-long prospective study of U.S. health professionals, indicates that individuals with a self-reported history of periodontal disease have a significantly elevated risk of developing HCC. This association remained robust after multivariate adjustment for confounders such as age, sex, smoking, alcohol consumption, obesity, and diabetes (34). These findings position oral dysbiosis as an independent risk factor for HCC and implicate *F. nucleatum* as an active contributor to HCC progression. Consequently, they provide a compelling rationale for targeting oral pathogens as a novel therapeutic avenue and source of prognostic biomarkers for HCC management.

## 3 Mechanisms of action: multi-pathway driven liver injury

### 3.1 Systemic translocation and direct action of bacteria

Oral bacteria are not restricted to the oral cavity. During routine activities such as chewing and brushing, or following dental procedures, oral microbes, particularly pathogenic species from periodontal pockets, can breach the compromised gingival epithelial barrier and enter the bloodstream, inducing transient bacteremia. The liver, which receives a dual blood supply from the portal vein and hepatic artery, functions as the primary filtration organ of the body and is consequently exposed to circulating oral pathogens. Upon reaching the hepatic sinusoids, these bacteria are typically phagocytosed and cleared by the resident Kupffer cells. However, certain species possessing specialized virulence factors can evade immune surveillance, persist within the liver, and directly interact with hepatocytes.

Studies have demonstrated that *F. nucleatum* utilizes its surface adhesin *FadA* to bind E-cadherin on hepatocytes. This ligand-receptor interaction triggers the activation of the Wnt/*β*-catenin signaling pathway, a core oncogenic cascade frequently dysregulated in HCC. Pathway activation drives the transcription of proto-oncogenes, such as c-Myc, thereby promoting aberrant hepatocyte proliferation and tumorigenesis (35). Furthermore, an additional adhesin, AldA, mediates bacterial attachment to the colonic epithelium. Following translocation via the oral–gut–liver axis, this adhesin may facilitate analogous hepatic colonization and proinflammatory mechanisms, indicating a broader multi-organ pathogenic capability for these microbes. The capacity for systemic translocation is not unique to *F. nucleatum* and *P. gingivalis.* Other periodontal pathogens, including *Aggregatibacter actinomycetemcomitans*, *Tannerella forsythia*, and *Treponema denticola*, have also been detected in the bloodstream following dental procedures or routine oral activities, suggesting that multiple oral bacterial species possess the potential to reach the liver and contribute to hepatic pathology (12). These bacteria may act synergistically or sequentially to promote liver injury through distinct yet complementary mechanisms.

### 3.2 Distal effects of microbial metabolites

The oral microbiota generates a diverse array of metabolites that, regardless of their beneficial or detrimental nature, can enter the systemic circulation and profoundly influence hepatic physiology. LPS, an endotoxin derived from the cell walls of oral gram-negative bacteria such as *P. gingivalis*, is a principal pathogenic factor. This molecule translocates into the bloodstream via compromised gingival vasculature and, upon reaching the liver, binds to TLR4 on Kupffer cells and hepatocytes. TLR4 engagement activates downstream pro-inflammatory cascades, notably NF-κB, which drives chronic hepatic inflammation, hepatic stellate cell activation, and fibrogenesis, which are key events in the progression of NASH and liver fibrosis (36).

Under dysbiotic conditions, periodontal pathogens like *P. gingivalis* degrade host proteins via proteolytic activity, generating substantial amounts of hydrogen sulfide (H₂S). Following systemic dissemination, H₂S suppresses cytochrome c oxidase in the hepatocyte mitochondrial electron transport chain. This interference impairs oxidative phosphorylation and exacerbates oxidative stress, thereby directly promoting hepatocyte injury and death (37). Beyond the effects of LPS and H₂S, periodontal pathogens contribute to hepatic injury through ROS-mediated mechanisms. The inflammatory response triggered by oral dysbiosis stimulates ROS generation from multiple cellular sources, including infiltrating neutrophils, activated Kupffer cells, and dysfunctional hepatocyte mitochondria. These ROS, in turn, act as signaling molecules that amplify the inflammatory cascade by activating the NF-κB pathway and the NLRP3 inflammasome, leading to sustained production of IL-1β, IL-6, and TNF-*α* (4). The resultant oxidative damage to hepatocyte DNA, proteins, and lipids further perpetuates the cycle of inflammation and tissue injury, establishing a feed-forward loop that drives the progression from simple steatosis to non-alcoholic steatohepatitis (NASH) and fibrosis (38). Importantly, the convergence of ROS-mediated oxidative damage and inflammation represents a common pathological denominator linking periodontitis, gut barrier dysfunction, and chronic liver disease. Furthermore, certain oral commensals, including specific *streptococci* and *Candida* species, possess alcohol-producing glycolytic pathways. In patients with NAFLD who abstain from alcohol, endogenous ethanol production represents a persistent source of hepatotoxic stress that may contribute to steatosis and inflammation (39).

However, not all microbial metabolites are deleterious. For example, oral *Actinomyces* such as *Rothia* species, can reduce dietary nitrate to nitrite, a precursor for systemic nitric oxide (NO) synthesis. NO confers beneficial effects by modulating hepatic perfusion and enhancing insulin sensitivity (40, 41). This functional duality underscores the complex and consequential role of the oral microbiome in maintaining systemic homeostasis.

### 3.3 Systemic immune-inflammation induced by oral dysbiosis

Oral bacteria, particularly periodontal pathogens, constitute more than a localized oral health concern. They contribute to systemic pathology through multiple synergistic mechanisms, including direct invasion, systemic inflammation induction, gut microbiota alteration, and host metabolism disruption.

Periodontitis is a chronic inflammatory disease that affects oral tissues and has consequences that extend beyond the oral cavity. The inflamed periodontal pocket continuously secretes a surfeit of pro-inflammatory cytokines, such as interleukin-1 beta (IL-1β), IL-6, and TNF-*α* into the circulation. Upon reaching the liver via the bloodstream, these cytokines establish systemic inflammation (42). Within the hepatic microenvironment, they activate resident Kupffer and hepatic stellate cells, directly exacerbating inflammatory responses and fibrotic processes, thereby fostering a critical microenvironment for the progression from NAFLD to NASH and advanced fibrosis (43). Furthermore, oral pathogens and their molecular components can promote systemic immune dysregulation, notably by driving the differentiation and activation of pro-inflammatory T helper 17 (Th17) cells, while potentially impairing regulatory T cell function. Th17 cells and their signature cytokine IL-17 can be recruited to the liver, where they amplify local inflammatory cascades. Through their actions on various hepatic cell populations, these factors collectively drive inflammation, fibrosis, and tumorigenesis (44). The interplay between ROS and immune activation is particularly relevant within the oral–gut–liver axis. Periodontal pathogens stimulate ROS production through activation of NADPH oxidases (NOX) in immune cells, particularly neutrophils and macrophages. The resulting oxidative stress not only inflicts direct tissue damage but also acts as a critical second messenger that amplifies inflammatory signaling (45). ROS activate the NLRP3 inflammasome, leading to caspase-1 activation and the maturation and secretion of IL-1β and IL-18—potent pro-inflammatory cytokines that exacerbate hepatic inflammation (46). Furthermore, ROS directly activate hepatic stellate cells, promoting their transdifferentiation into myofibroblasts and thereby driving liver fibrogenesis (47). Thus, periodontal pathogen-induced ROS generation represents a key mechanistic link between oral inflammation and liver pathology.

Given the complexity of the hepatic immune microenvironment, NK cells and T cells—as core effector cells mediating innate and adaptive immune responses *in situ*—play an essential role in responding to microbial signals and inflammatory mediators originating from oral dysbiosis, thereby serving as a critical link between local oral infection and systemic immunopathological injury in the liver (33). The involvement of NK and T cells underscores their central roles in hepatic immunity, as these cells actively respond to microbial signals and inflammatory mediators originating from oral dysbiosis, thereby contributing to the regulation of liver inflammation, fibrogenesis, and antitumor immune surveillance within the oral–gut–liver axis.

### 3.4 Gut microbiota-mediated indirect pathway

The gut microbiota-mediated indirect pathway is a major focus of current research. An animal study demonstrated that oral administration of *P. gingivalis* alters the gut microbiota composition in mice, concomitant with changes in insulin resistance and gene expression in the adipose tissue and liver, indicating complex crosstalk between the oral and gut microbial communities (48). Oral bacteria are continuously ingested with saliva, constituting a major exogenous source for the gut ecosystem and providing a route for intestinal translocation. Once established, these bacteria subsequently influence host physiology, including bile acid metabolism. A 2020 study found that oral-derived *Streptococcus mutans* in the gut can convert conjugated bile acids to free bile acids via bile salt hydrolase activity, resulting in the inhibition of the hepatic FXR signaling pathway. The accumulation of free bile acids inhibits FXR, downregulates cholesterol 7α-hydroxylase (CYP7A1), and consequently impedes bile acid synthesis (49). Under specific pathological conditions, such as liver disease, an altered intestinal environment may enable certain oral bacteria to evade gastric acid and intestinal barrier defenses. This facilitates their translocation and subsequent aberrant colonization within the intestine, ultimately reshaping the gut microbiota composition toward a state enriched with orally derived taxa, a shift often described as microbial “oralization” (15). These aberrantly colonizing oral species secrete virulence factors, including proteases, which disrupt intestinal epithelial tight junctions and increase intestinal permeability. A compromised barrier function permits a substantial influx of gut-derived bacterial products into the liver via portal circulation. This exposes hepatic Kupffer and other immune cells to sustained, high-level microbial stimulation, triggering a more intense and persistent inflammatory response that drives chronic liver injury, fibrosis, and hepatocarcinogenesis (50). Consequently, salivary analysis could emerge as a simple, rapid, and non-invasive clinical tool for assessing the gut ecosystem status and systemic disease burden in patients with cirrhosis, offering a novel perspective for liver disease management.

## 4 Translational medicine and clinical prospects

### 4.1 The oral microbiome as diagnostic and prognostic biomarkers

Saliva and dental plaque samples are promising candidates for disease screening and monitoring because of their noninvasive and easily accessible nature. Clinical evidence indicates that the oral microbiome can serve as an effective noninvasive biomarker capable of distinguishing patients with liver disease from healthy controls and facilitating longitudinal monitoring of disease progression. For instance, a metagenomic sequencing analysis of saliva samples identified characteristic alterations in the salivary microbiota of patients with cirrhosis and constructed a diagnostic model whose accuracy was comparable to that of models based on the fecal microbiome (51). Research on NAFLD further reveals that the relative abundance of specific bacterial taxa in the salivary microbiota correlates with the degree of hepatic steatosis and stage of fibrosis, suggesting its potential utility for stratifying NAFLD severity. In the context of HCC, the detection of *F. nucleatum* within tumor tissue has been established as an independent predictor of poor prognosis, indicating that patients harboring this bacterium typically experience shorter overall and recurrence-free survival (52).

While current detection methods rely on tissue biopsies, a critical future direction involves identifying circulating microbial or immune markers in blood or saliva associated with intratumoral *F. nucleatum*, thereby enabling non-invasive prognostic assessment.

### 4.2 Oral health intervention: a novel adjunctive management strategy for liver disease

A core question in translational research is whether improving oral health, particularly through periodontitis treatment, can positively influence liver disease progression. Preliminary clinical intervention studies have provided encouraging evidence. The preventive role of oral care in serious complications of cirrhosis is gaining recognition. Studies have indicated that a patient’s oral hygiene status is independently associated with the risk of hepatic encephalopathy. Furthermore, intervention trials have demonstrated that implementing intensive oral care, including professional periodontal cleaning and daily chlorhexidine mouthwash use, in patients with decompensated cirrhosis can significantly reduce systemic inflammatory markers and show a trend toward decreased hospitalization rates. This directly supports the beneficial effects of periodontal treatment on liver-related metabolism and inflammation (53, 54). A randomized controlled trial specifically showed that non-surgical periodontal therapy in patients with cirrhosis not only improved their oral health but also beneficially modulated the oral–gut–liver axis by improving gut microbiota profiles, reducing systemic inflammation, and enhancing cognitive function related to hepatic encephalopathy. This provides strong interventional evidence for incorporating periodontal therapy into comprehensive cirrhosis management (53). Although larger prospective randomized controlled trials (RCTs) are warranted for confirmation, these findings underscore the importance of integrating oral care into multidisciplinary management of cirrhosis. From a pragmatic standpoint, oral interventions, such as basic periodontal therapy, are mature, safe, and relatively low-cost clinical procedures. Their integration as a routine adjunctive strategy for patients with liver disease is highly feasible and offers favorable cost-effectiveness.

Such interventions are not intended to replace established liver therapies but serve as a complementary approach capable of improving systemic inflammatory status, potentially slowing disease progression, and securing a role in the early prevention and holistic management of liver disease.

### 4.3 Towards precision medicine

Future therapeutic strategies targeting the oral microbiota may evolve beyond conventional periodontal treatment toward more precise and targeted modulations. The development of specific interventions against key oral pathogens encompasses several strategies. First, the use of targeted agents, such as bacteriophages or narrow-spectrum antimicrobials, could eliminate specific pathogens while preserving the ecological balance of the broader microbial community. Second, vaccines or monoclonal antibodies directed against these pathogens could prevent their initial colonization or neutralize their virulence factors. Third, probiotic or prebiotic interventions involving the administration of beneficial oral bacterial strains or substrates that selectively promote their growth to competitively inhibit pathogenic expansion. These approaches are grounded in the understanding that systemic health relies on a sophisticated and dynamic symbiosis between the host and its microbiome. Disruption of this equilibrium is a central mechanism driving a spectrum of immune-mediated, metabolic, and neoplastic diseases (55).

An emerging concept is oral microbiota transplantation (OMT), which involves resetting a patient’s dysbiotic flora using microbiota from a healthy donor. While promising in animal models of caries and periodontitis, its applicability in liver disease requires substantial further investigation. Ultimately, integrating patient-derived multi-omics data, including metagenomic, metabolomic, and host genetic profiles, will enable precise stratification of dysregulation along the oral–gut–liver axis. This paves the way for personalized interventions, such as prioritizing periodontal management for patients with elevated systemic LPS and considering targeted pathogen eradication in HCC patients harboring *F. nucleatum.*

## 5 Challenges, controversies, and future directions

### 5.1 Current methodological challenges

Although substantial evidence supports the concept of the “oral–gut–liver axis,” this field remains in a phase of rapid development, facing numerous methodological challenges, scientific controversies, and unresolved questions. Addressing these challenges and strategically defining future research directions is pivotal for advancing the field from correlative observations toward mechanistic understanding and, ultimately, clinical translation of these findings. Method heterogeneity considerably limits the reliability and comparability of the research findings. Studies utilize diverse sample types, including saliva, gingival crevicular fluid, buccal swabs, and dental plaque, whose microbial compositions vary significantly and are highly sensitive to collection timing, diet, and oral hygiene practices, thereby complicating direct cross-study comparisons (56). Furthermore, technical choices related to 16S rRNA gene sequencing primers, sequencing depth, and the selection of library preparation and sequencing platforms for metagenomics can introduce bias in microbial profiling and functional inferences. Much of this variability originates from differences in sample processing, DNA extraction protocols, and sequencing facilities, with bioinformatics pipelines playing a comparatively smaller, albeit non-negligible, role (57). The absence of a gold-standard bioinformatics workflow further exacerbates these inconsistencies. Most existing evidence is derived from observational studies, which cannot fully account for residual confounding by factors such as detailed dietary patterns and socioeconomic status, nor can they definitively establish causal direction. Although Mendelian randomization studies offer insights into potential causality, their validity depends on the strength of the instrumental variables used and may be influenced by pleiotropic effects (25).

### 5.2 Key unanswered questions

Current research on the oral microbiome in liver disease remains largely restricted to the genus and species levels. However, the virulence properties and functional impacts can vary substantially among different strains within a single species. For instance, not all strains of *F. nucleatum* harbor the same oncogenic potential. Therefore, future investigations must integrate metagenomics with culturomics to identify specific virulent strains and their genetic determinants that actively drive hepatic pathology, thereby advancing the field toward a more nuanced strain-level understanding. This underscores the need to extend analytical resolution to the strain level, incorporating longitudinal multi-omics data, to accurately decipher the influence of the microbiome on health. This approach establishes a critical foundation for developing microbiome-based precision diagnostics and dynamic monitoring tools (58).

Furthermore, existing research exhibits a strong bias toward bacterial components (bacteriomes). The oral cavity harbors a diverse consortium of fungi (e.g., *Candida*) and viruses (including bacteriophages and eukaryotic viruses), which interact with bacterial communities to shape the oral ecosystem. The specific contributions of these non-bacterial members to liver disease pathogenesis remain largely uncharted. For example, while the role of oral *Candida* in alcoholic liver disease is beginning to be recognized, its involvement in NAFLD or HCC remains unclear (59).

The four major mechanistic pathways (summarized in Table 1) outlined in this review are not mutually exclusive. They likely operate synergistically or sequentially, depending on individual host factors, specific liver disease, and its stage. However, the relative weight of each pathway, their complex interplay, and the host genetic or environmental triggers that favor one pathway over another, remain poorly characterized. Consequently, future research must employ integrated multi-omics frameworks, including strain-resolved metagenomics, to concurrently capture fungal and viral components. This comprehensive strategy is indispensable for dynamically unraveling the interactions within the network of pathogenic circuits.

**Table 1:** Summary of core pathogenic mechanisms of the oral–gut–liver axis.

Mechanisms	Descriptions
Direct bacterial translocation	Oral pathogens (e.g., *P. gingivalis*, *F. nucleatum*, *A. actinomycetemcomitans*, *T. denticola*) hematogenously seed the liver and directly activate oncogenic pathways in hepatocytes (32, 35).
Metabolite-mediated toxicity	Circulating microbial products trigger hepatic inflammation and oxidative stress via immune cell activation (36, 37, 39).
Systemic immune-inflammatory activation	The periodontium functions systemically releasing cytokines that exacerbate liver inflammation and fibrosis (42, 43).
Gut microbiota-mediated indirect effects	Ingested oral bacteria disrupt gut ecology and barrier integrity, increasing gut-derived toxins and aggravating liver injury (48–50).

**Figure 1:**
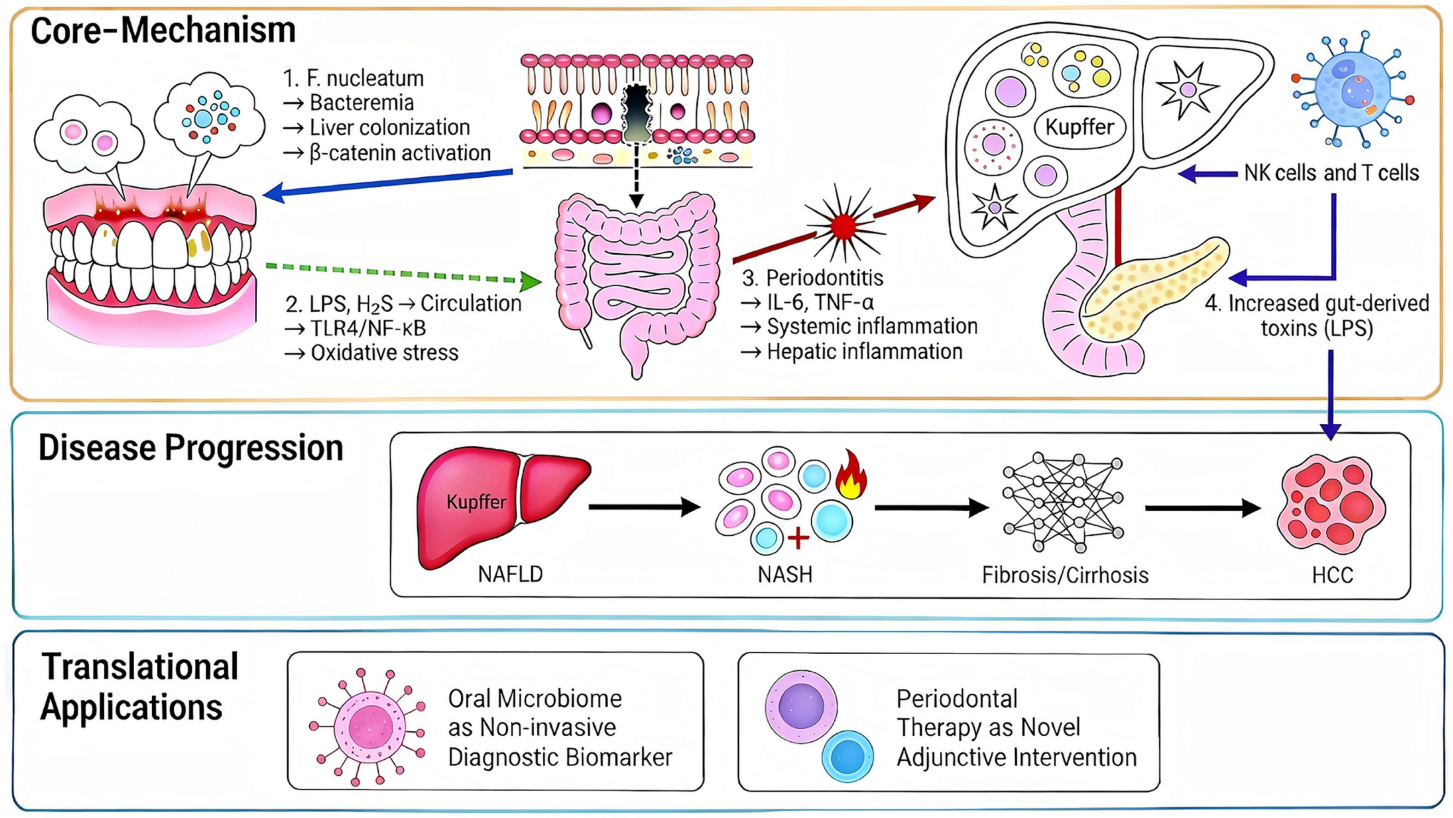
Schematic overview of the oral–gut–liver axis in liver disease pathogenesis and its translational implications. LPS, lipopolysaccharide; TLR4, Toll-like receptor 4; NF-κB, nuclear factor kappa B; IL, interleukin; TNF-*α*, tumor necrosis factor-alpha; NAFLD, non-alcoholic fatty liver disease; NASH, non-alcoholic steatohepatitis; HCC, hepatocellular carcinoma.

The four interconnected pathways outlined in Table 1 collectively mediate the impact of oral dysbiosis on the liver. Their spatial relationships, interactive dynamics, and clinical consequences are visually synthesized in Figure 1.

### 5.3 Future research roadmap

To overcome these challenges and address core scientific questions, a coordinated and multifaceted research strategy is imperative. Future studies must integrate resources from hepatology and oral medicine centers to prospectively collect standardized, multi-type biospecimens, coupled with comprehensive clinical phenotyping, lifestyle, and dietary data. This foundation is essential for generating reproducible findings and enabling rigorous mechanistic and translational studies. Experimental models should include colonizing germ-free or antibiotic-treated mice with patient-derived oral or gut microbiota, which is a gold-standard approach for establishing microbial causality. The integration of gene-editing technologies will further allow for the specific study of host gene-microbe interactions.

An emerging direction involves employing advanced *in vitro* systems, such as liver organoids or multi-tissue “gut-liver” chip models, which permit the precise dissection of the direct effects of specific oral bacteria or metabolites on hepatocytes and the associated immune responses, leveraging a highly controlled microenvironment (60). Based on these mechanistic insights, clinical translation must be actively pursued. This necessitates the design and implementation of multicenter, large-sample randomized controlled trials (RCTs) to evaluate the long-term impact of systematic periodontal therapy or other oral interventions on hard histological liver endpoints, HCC incidence, and cirrhosis-related complications. Ultimately, progress in this field fundamentally relies on close collaboration across disciplines, including hepatology, oral medicine, microbiology, immunology, bioinformatics, and public health. Establishing sustained interdisciplinary consortia and integrated research teams is crucial for synthesizing knowledge, technologies, and methodologies, thereby realizing the complete translational pathway from bench to bedside and into broader community health practices.

## 6 Conclusion

In summary, converging evidence from multiple dimensions substantiates the pivotal role of the “oral–gut–liver axis” in the pathophysiology of liver diseases. Oral microbiota is not an isolated ecosystem; its dysbiosis remotely drives hepatic inflammation, fibrosis, and carcinogenesis through several parallel and interconnected pathways, including direct bacterial translocation, metabolite-mediated toxicity, systemic immune-inflammatory activation, and gut microbiota-mediated indirect effects. Epidemiological studies have established oral dysbiosis, particularly periodontitis, as an independent and modifiable risk factor for NAFLD and HCC. This scientific consensus carries significant public health implications, revealing that maintaining oral health extends beyond the prevention of local caries and periodontal disease; it represents a low-cost, noninvasive strategy with considerable potential for the primary prevention and adjunctive management of chronic liver disease. Given the escalating global burden of liver disease and the persistent limitations of specific therapies, leveraging the “oral–liver axis” as a novel preventive approach offers high cost-effectiveness and accessibility. Consequently, the evidence supporting the systematic integration of oral health assessment and intervention into the clinical management and public health strategy for liver diseases is now robust. Future research priorities should focus on delineating the interactive network of the oral–gut–liver microbiota, developing dynamic multi-bodily-fluid biomarkers, verifying the causal chain linking periodontal therapy to gut microbiota remodeling and subsequent hepatic inflammation alleviation, and ultimately facilitating the clinical implementation of precision prevention strategies.

## References

[1] Younossi ZM Koenig AB Abdelatif D Fazel Y Henry L Wymer M. Global epidemiology of nonalcoholic fatty liver disease-meta-analytic assessment of prevalence, incidence, and outcomes. Hepatology. (2016) 64:73–84. doi: 10.1002/hep.28431, 26707365

[2] Verma M Younossi Z. Integrating patient-reported outcomes within routine hepatology care: a prompt to action. Hepatology. (2021) 73:1570–80. doi: 10.1002/hep.31550, 32918286

[3] Singal AG Lampertico P Nahon P. Epidemiology and surveillance for hepatocellular carcinoma: new trends. J Hepatol. (2020) 72:250–61. doi: 10.1016/j.jhep.2019.08.025, 31954490 PMC6986771

[4] Renu K Gopalakrishnan AV Madhyastha H. Is periodontitis triggering an inflammatory response in the liver, and does this reaction entail oxidative stress? Odontology. (2025) 113:889–902. doi: 10.1007/s10266-024-01032-x, 39621235

[5] Jyoti DP. Mechanisms and implications of the gut microbial modulation of intestinal metabolic processes. NPJ Metab Health Dis. (2025) 3:24. doi: 10.1038/s44324-025-00066-1 40604123 PMC12441142

[6] Avila M Ojcius DM Yilmaz O. The oral microbiota: living with a permanent guest. DNA Cell Biol. (2009) 28:405–11. doi: 10.1089/dna.2009.0874, 19485767 PMC2768665

[7] Aberg F Helenius-Hietala J. Oral health and liver disease: bidirectional associations-a narrative review. Dent J (Basel). (2022) 10:16. doi: 10.3390/dj10020016, 35200242 PMC8870998

[8] Lee S Haraga H Satoh T Mutoh N Watanabe K Hamada N . Effect of periodontitis induced by *Fusobacterium nucleatum* on the microbiota of the gut and surrounding organs. Odontology. (2024) 112:177–84. doi: 10.1007/s10266-023-00827-8, 37432500

[9] Engevik MA Danhof HA Ruan W . *Fusobacterium nucleatum* secretes outer membrane vesicles and promotes intestinal inflammation. MBio. (2021) 12:e2706–20. doi: 10.1128/mBio.02706-20 PMC8092269 33653893

[10] Nagao Y Tanigawa T. Red complex periodontal pathogens are risk factors for liver cirrhosis. Biomed Rep. (2019) 11:199–206. doi: 10.3892/br.2019.1245, 31632667 PMC6792321

[11] Komazaki R Katagiri S Takahashi H Maekawa S Shiba T Takeuchi Y . Periodontal pathogenic bacteria, *Aggregatibacter actinomycetemcomitans* affect non-alcoholic fatty liver disease by altering gut microbiota and glucose metabolism. Sci Rep. (2017) 7:13950. doi: 10.1038/s41598-017-14260-9, 29066788 PMC5655179

[12] Dioguardi M Lo Muzio E Guerra C Sovereto D Laneve E Martella A . Liver disease and periodontal pathogens: a bidirectional relationship between liver and Oral microbiota. Dent J. (2025) 13:503. doi: 10.3390/dj13110503, 41294484 PMC12651693

[13] Yue Y Liu X Li Y . The role of TLR4/MyD88/NF-kappaB pathway in periodontitis-induced liver inflammation of rats. Oral Dis. (2021) 27:1012–21. doi: 10.1111/odi.13616 32853444 PMC8247295

[14] Acharya C Sahingur SE Bajaj JS. Microbiota, cirrhosis, and the emerging oral-gut-liver axis. JCI Insight. (2017) 2. doi: 10.1172/jci.insight.94416, 28978799 PMC5841881

[15] Bajaj JS Betrapally NS Hylemon PB Heuman DM Daita K White MB . Salivary microbiota reflects changes in gut microbiota in cirrhosis with hepatic encephalopathy. Hepatology. (2015) 62:1260–71. doi: 10.1002/hep.27819, 25820757 PMC4587995

[16] Yoneda M Naka S Nakano K Wada K Endo H Mawatari H . Involvement of a periodontal pathogen, *Porphyromonas gingivalis* on the pathogenesis of non-alcoholic fatty liver disease. BMC Gastroenterol. (2012) 12:16. doi: 10.1186/1471-230X-12-16, 22340817 PMC3305584

[17] Kuraji R Sekino S Kapila Y Numabe Y. Periodontal disease-related nonalcoholic fatty liver disease and nonalcoholic steatohepatitis: an emerging concept of oral-liver axis. Periodontol. (2021) 87:204–40. doi: 10.1111/prd.12387, 34463983 PMC8456799

[18] Idrissi Janati A Karp I Von Renteln D Bouin M Liu Y Tran SD . Investigation of *Fusobacterium nucleatum* in saliva and colorectal mucosa: a pilot study. Sci Rep. (2022) 12:5622. doi: 10.1038/s41598-022-09587-x, 35379861 PMC8979950

[19] Gao Y Zhang P Wei Y Ye C Mao D Xia D . *Porphyromonas gingivalis* exacerbates alcoholic liver disease by altering gut microbiota composition and host immune response in mice. J Clin Periodontol. (2023) 50:1253–63. doi: 10.1111/jcpe.13833 37381658

[20] Han P Sun D Yang J. Interaction between periodontitis and liver diseases. Biomed Rep. (2016) 5:267–76. doi: 10.3892/br.2016.718, 27588170 PMC4998044

[21] Singh S Allen AM Wang Z Prokop LJ Murad MH Loomba R. Fibrosis progression in nonalcoholic fatty liver vs nonalcoholic steatohepatitis: a systematic review and Meta-analysis of paired-biopsy studies. Clin Gastroenterol Hepatol. (2015) 13:643–654.e9. doi: 10.1016/j.cgh.2014.04.014, 24768810 PMC4208976

[22] Muthiah MD Cheng Han N Sanyal AJ. A clinical overview of non-alcoholic fatty liver disease: a guide to diagnosis, the clinical features, and complications-what the non-specialist needs to know. Diabetes Obes Metab. (2022) 24:3–14. doi: 10.1111/dom.14521, 34387409

[23] Huh Y Cho YJ Nam GE. Recent epidemiology and risk factors of nonalcoholic fatty liver disease. J Obes Metab Syndr. (2022) 31:17–27. doi: 10.7570/jomes22021, 35332111 PMC8987457

[24] Qiao F Fu K Zhang Q Liu L Meng G Wu H . The association between missing teeth and non-alcoholic fatty liver disease in adults. J Clin Periodontol. (2018) 45:941–51. doi: 10.1111/jcpe.12929, 29779210

[25] Qiao F Li X Liu Y Zhang S Liu D Li C. Periodontitis and NAFLD-related diseases: a bidirectional two-sample mendelian randomization study. Oral Dis. (2024) 30:3452–61. doi: 10.1111/odi.14785, 37877540

[26] Hudson D Ayares G Taboun Z Malhi G Idalsoaga F Mortuza R . Periodontal disease and cirrhosis: current concepts and future prospects. eGastroenterology. (2025) 3:e100140. doi: 10.1136/egastro-2024-100140, 40160254 PMC11950965

[27] Rodríguez-Montaño R Martínez-Nieto M González-Alvarez GE Alarcón-Sánchez MA Becerra-Ruiz JS Heboyan A . Hepatitis and periodontal health: an emerging oral-liver axis. Ther Adv Chronic Dis. (2025) 16:404416666. doi: 10.1177/20406223251368090, 40851818 PMC12368331

[28] Gronkjaer LL. Periodontal disease and liver cirrhosis: a systematic review. SAGE Open Med. (2015) 3:2102673122. doi: 10.1177/2050312115601122, 26770799 PMC4679327

[29] Kuraji R Shiba T Dong TS Numabe Y Kapila YL. Periodontal treatment and microbiome-targeted therapy in management of periodontitis-related nonalcoholic fatty liver disease with oral and gut dysbiosis. World J Gastroenterol. (2023) 29:967–96. doi: 10.3748/wjg.v29.i6.967, 36844143 PMC9950865

[30] Ren Z Li A Jiang J Zhou L Yu Z Lu H . Gut microbiome analysis as a tool towards targeted non-invasive biomarkers for early hepatocellular carcinoma. Gut. (2019) 68:1014–23. doi: 10.1136/gutjnl-2017-315084, 30045880 PMC6580753

[31] Datorre JG Dos Reis MB Sorroche BP Teixeira GR Hatano SS de Carvalho AC . Intratumoral *Fusobacterium nucleatum* is associated with better cancer-specific survival in head and neck cancer patients. J Oral Microbiol. (2025) 17:2487644. doi: 10.1080/20002297.2025.2487644, 40182114 PMC11966973

[32] Ye C Liu X Liu Z Pan C Zhang X Zhao Z . *Fusobacterium nucleatum* in tumors: from tumorigenesis to tumor metastasis and tumor resistance. Cancer Biol Ther. (2024) 25:2306676. doi: 10.1080/15384047.2024.2306676, 38289287 PMC10829845

[33] Gur C Ibrahim Y Isaacson B Yamin R Abed J Gamliel M . Binding of the Fap2 protein of *Fusobacterium nucleatum* to human inhibitory receptor TIGIT protects tumors from immune cell attack. Immunity. (2015) 42:344–55. doi: 10.1016/j.immuni.2015.01.010, 25680274 PMC4361732

[34] Michaud DS Kelsey KT Papathanasiou E Genco CA Giovannucci E. Periodontal disease and risk of all cancers among male never smokers: an updated analysis of the health professionals follow-up study. Ann Oncol. (2016) 27:941–7. doi: 10.1093/annonc/mdw028, 26811350 PMC4843185

[35] Rubinstein MR Wang X Liu W Hao Y Cai G Han YW. *Fusobacterium nucleatum* promotes colorectal carcinogenesis by modulating E-cadherin/β-catenin signaling via its FadA adhesin. Cell Host Microbe. (2013) 14:195–206. doi: 10.1016/j.chom.2013.07.012, 23954158 PMC3770529

[36] Henao-Mejia J Elinav E Jin C Hao L Mehal WZ Strowig T . Inflammasome-mediated dysbiosis regulates progression of NAFLD and obesity. Nature. (2012) 482:179–85. doi: 10.1038/nature10809, 22297845 PMC3276682

[37] Mateus I Prip-Buus C. Hydrogen sulphide in liver glucose/lipid metabolism and non-alcoholic fatty liver disease. Eur J Clin Investig. (2022) 52:e13680. doi: 10.1111/eci.13680, 34519030 PMC9285505

[38] Dornas W Schuppan D. Mitochondrial oxidative injury: a key player in nonalcoholic fatty liver disease. Am J Physiol Gastrointest Liver Physiol. (2020) 319:G400–11. doi: 10.1152/ajpgi.00121.2020, 32597705

[39] Zhu L Baker SS Gill C Liu W Alkhouri R Baker RD . Characterization of gut microbiomes in nonalcoholic steatohepatitis (NASH) patients: a connection between endogenous alcohol and NASH. Hepatology. (2013) 57:601–9. doi: 10.1002/hep.26093 23055155

[40] Feng J Liu J Jiang M Chen Q Zhang Y Yang M . The role of oral nitrate-reducing bacteria in the prevention of caries: a review related to caries and nitrate metabolism. Caries Res. (2023) 57:119–32. doi: 10.1159/000529162, 36649690

[41] Kus E Jasinski K Skorka T . Short-term treatment with hepatoselective NO donor V-PYRRO/NO improves blood flow in hepatic microcirculation in liver steatosis in mice. Pharmacol Rep. (2018) 70:463–9. doi: 10.1016/j.pharep.2017.11.019, 29631249

[42] Demmer RT Squillaro A Papapanou PN Rosenbaum M Friedewald WT Jacobs DR Jr . Periodontal infection, systemic inflammation, and insulin resistance: results from the continuous National Health and Nutrition Examination Survey (NHANES) 1999-2004. Diabetes Care. (2012) 35:2235–42. doi: 10.2337/dc12-0072, 22837370 PMC3476901

[43] Mei E Yao C Chen Y Mei E-H Chen Y-N Nan S-X . Multifunctional role of oral bacteria in the progression of non-alcoholic fatty liver disease. World J Hepatol. (2024) 16:688–702. doi: 10.4254/wjh.v16.i5.688, 38818294 PMC11135273

[44] Li N Yamamoto G Fuji H Kisseleva T. Interleukin-17 in liver disease pathogenesis. Semin Liver Dis. (2021) 41:507–15. doi: 10.1055/s-0041-1730926, 34130335

[45] Ramos-Tovar E Muriel P. Molecular mechanisms that link oxidative stress, inflammation, and fibrosis in the liver. Antioxidants. (2020) 9:1279. doi: 10.3390/antiox9121279, 33333846 PMC7765317

[46] Kabacaoğlu B Öztürk Özener H. Evaluation of inflammasomes as biomarker following non-surgical periodontal treatment. Arch Oral Biol. (2024) 164:105987. doi: 10.1016/j.archoralbio.2024.105987 38723420

[47] Gandhi CR. Oxidative stress and hepatic stellate cells: a paradoxical relationship. Trends Cell Mol Biol. (2012) 7:1–10. 27721591 PMC5051570

[48] Arimatsu K Yamada H Miyazawa H Minagawa T Nakajima M Ryder MI . Oral pathobiont induces systemic inflammation and metabolic changes associated with alteration of gut microbiota. Sci Rep. (2014) 4:4828. doi: 10.1038/srep04828, 24797416 PMC4010932

[49] Kitamoto S Nagao-Kitamoto H Jiao Y Gillilland MG III Hayashi A Imai J . The intermucosal connection between the mouth and gut in commensal pathobiont-driven colitis. Cell. (2020) 182:447–462.e14. doi: 10.1016/j.cell.2020.05.048, 32758418 PMC7414097

[50] Tilg H Adolph TE Trauner M. Gut-liver axis: pathophysiological concepts and clinical implications. Cell Metab. (2022) 34:1700–18. doi: 10.1016/j.cmet.2022.09.017, 36208625

[51] Oh TG Kim SM Caussy C Fu T Guo J Bassirian S . A universal gut-microbiome-derived signature predicts cirrhosis. Cell Metab. (2020) 32:e6:878–88. doi: 10.1016/j.cmet.2020.06.005, 32610095 PMC7822714

[52] Yamamura K Baba Y Nakagawa S Mima K Miyake K Nakamura K . Human microbiome *Fusobacterium nucleatum* in esophageal cancer tissue is associated with prognosis. Clin Cancer Res. (2016) 22:5574–81. doi: 10.1158/1078-0432.CCR-16-1786 27769987

[53] Bajaj JS Matin P White MB Fagan A Deeb JG Acharya C . Periodontal therapy favorably modulates the oral-gut-hepatic axis in cirrhosis. Am J Physiol Gastrointest Liver Physiol. (2018) 315:G824–37. doi: 10.1152/ajpgi.00230.2018, 30118351 PMC6293251

[54] Kwon T Lamster IB Levin L. Current concepts in the management of periodontitis. Int Dent J. (2021) 71:462–76. doi: 10.1111/idj.12630, 34839889 PMC9275292

[55] Zheng D Liwinski T Elinav E. Interaction between microbiota and immunity in health and disease. Cell Res. (2020) 30:492–506. doi: 10.1038/s41422-020-0332-7, 32433595 PMC7264227

[56] Hall MW Singh N Ng KF Lam DK Goldberg MB Tenenbaum HC . Inter-personal diversity and temporal dynamics of dental, tongue, and salivary microbiota in the healthy oral cavity. NPJ Biofilms Microbiomes. (2017) 3:2. doi: 10.1038/s41522-016-0011-0, 28649403 PMC5445578

[57] Sinha R Abu-Ali G Vogtmann E Fodor AA Ren B Amir A . Assessment of variation in microbial community amplicon sequencing by the microbiome quality control (MBQC) project consortium. Nat Biotechnol. (2017) 35:1077–86. doi: 10.1038/nbt.3981, 28967885 PMC5839636

[58] Lloyd-Price J Mahurkar A Rahnavard G Crabtree J Orvis J Hall AB . Strains, functions and dynamics in the expanded human microbiome project. Nature. (2017) 550:61–6. doi: 10.1038/nature23889, 28953883 PMC5831082

[59] Yang A Inamine T Hochrath K Yang A-M Chen P Wang L . Intestinal fungi contribute to development of alcoholic liver disease. J Clin Invest. (2017) 127:2829–41. doi: 10.1172/JCI90562, 28530644 PMC5490775

[60] Kostrzewski T Cornforth T Snow SA Ouro-Gnao L Rowe C Large EM . Three-dimensional perfused human in vitro model of non-alcoholic fatty liver disease. World J Gastroenterol. (2017) 23:204–15. doi: 10.3748/wjg.v23.i2.204, 28127194 PMC5236500

